# Correction: Within-subjects ultra-short sleep-wake protocol for characterising circadian variations in retinal function

**DOI:** 10.1371/journal.pone.0336258

**Published:** 2025-11-04

**Authors:** The PLOS One Staff

In the abstract section there is an error in the fifth sentence. Participants (n = 12, 50% female) will stay in a controlled time-isolating environment under dim-light conditions and adhere to an ultra-short sleep-wake cycle, alternating between 2 hours and 30 minutes (150 minutes) of wake time in dim light, and 1 hour and 15 minutes (75 minutes) of sleep in darkness, maintaining a sleep-wake ratio of 2:1 during each sleep-wake cycle.

The publisher apologizes for the error.

## References

[1] Heinrichs HS, Spitschan M. Within-subjects ultra-short sleep-wake protocol for characterising circadian variations in retinal function. PLoS One. 2025;20(5):e0300405. doi: 10.1371/journal.pone.0300405 40373053 PMC12080756

