# Supplementary material for: Within-subjects ultra-short sleep-wake protocol for characterising circadian variations in retinal function
Source: PLoS One. 2025 May 15;20(5):e0300405. doi: 10.1371/journal.pone.0300405 (PMC12080756; doi:10.1371/journal.pone.0300405)
Supplement: Attachment — Submitted filename: 20240916_revision-1_answers.pdf [file pone.0300405.s001.pdf]

Editor Comments: Thank you for your submission. Your manuscript has been reviewed and the reviewers are enthusiastic about the manuscript. However, they also raise important points, especially around methodology, rationale, and terminology. I ask that you please give these careful consideration and revise the manuscript accordingly.

We thank the editor for their comment and for the opportunity to revise the manuscript. We have revised the manuscript following the reviewers' comments. We respond to each reviewer comment below.

Reviewer 1

The study protocol from Heinrichs and Spitschan is carefully well-designed It deals with a major question in the intersection of vision and circadian science, which is whether circadian variation in human physiology has a consequence in early light information processing. They focus on vision sensitivity and pupillometry to study this potential effect.

In the following text, I include my comments on the manuscript starting with the major concerns.

[R1.1] The hypotheses of this study are based on the selective and independent stimulation of each post-receptoral pathway (L+M+S, L-M, and S) for both pupillometry and psychophysics. To achieve this selectivity, the authors will use the silent substitution method. However, this method can be affected by individual variability. The authors recognize this weakness (Line 717), but they claim that it shouldn't be a problem with high-contrast stimulation. With the devices that they are going to use, they might achieve a good contrast for L+M+S and possibly S. However, L-M contrast is usually very low with any device, especially when controlling melanopsin. The authors should include an individual calibration procedure and report what are the possible contrasts for each stimulation condition with their devices. This is very important since the main outcomes of the study are based on this selectivity.

We thank the reviewer for this comment and clarify the following:

The two devices (CRS Metropsis Research system and custom binocular Maxwellian-view pupillometer) are equipped with different primaries, and therefore, both the maximum contrasts and the effective contrasts differ between the psychophysical and the pupillometric assessment. Additionally, the Metropsis Research display system uses RGB rather than the multi-primary system used in the pupillometer. In the pupillometric measurement, contrasts were ±30% against the background spectrum that was produced by all LEDs being at half maximum intensity. In the psychophysical assessment, we utilized the maximum contrast possible given the RGB colour gamut of the monitor ([L+M+S]: 100%, [L-M]: 6%, and [S]: 51%) with lower starting values were partly lower as a trade-off between experiment length and visibility of the stimuli ([L+M+S]: 90%, [L-M]: 6%, and [S]: 35%).

Regarding the calibration, the CRS Metropsis Research system is calibrated by the distributor, and at setup, the NVBL calibration procedure is part of the stimulus calculation and is described in the supplementary material.

We take the point that individual calibrations can increase the precision of stimulation. Given the time constraints in this protocol, we deemed individual observer calibration procedures infeasible given time restrictions and the use of naive participants (unlike highly experienced psychophysical observers). After the study concludes, we will include a simulation calculating the residual variance in the effective contrast experienced by the observers resulting from variation in idiosyncratic particularities in retinal physiology. The procedure used for this will follow the splatter calculation logic developed by Spitschan et al. (2015), in which the contrast seen by variant observers is calculated over a range of parameter choices.

[R1.2] The hypotheses are a little poorly detailed. Based on the literature, the authors might want to include what they expect. For example, Zele and colleagues found that melanopsin-driven contribution is higher in afternoon hours (Zele et al., Plos One, 2011). Do the authors think that something similar or contrary will be expected in their study?

We are stating an open hypothesis for circadian rhythmicity in (post)-receptoral components of the retina. Using the method of silent substitution, we are able to stimulate the photoreceptors in a selective fashion, enabling the disentanglement of melanopsin- and cone-mediated circadian rhythmicity. Of course, the prior studies cited by the reviewer indicate that there appears to be some circadian rhythmicity in the pupil response to monochromatic lights (using the "PIPR" paradigm), but these

measurements did not allow for the clean separation of melanopsin- vs. cone-mediated contributions. It is clear that the paradigms used are very different and that our study is complementary to the existing literature.

Specifically, we believe that this study adds valuable new insights to the body of literature. As detailed in the manuscript, this is particularly the case as it uniquely offers the opportunity to minimize the effects of homeostasis, especially sleep pressure, satiety, etc. Furthermore, very few research groups employ the method of silent substitution for their stimuli to achieve the selective isolation of specific photoreceptor classes and/or opponency and additive processes.

While a replication would add to the reliability of previous findings, a failure to replicate (specifically, but also conceptually) would inform the limitations of either publication's methods.

We have elaborated slightly in the manuscript to emphasize the novelty of this study compared to previous literature:

> *"To what extent the PRC is driven by a modulation of sensitivity in the retina or mechanisms in the SCN is unknown. Studies investigating how the pupillary light reflex, which reflects the response of all photoreceptors to light [12, 13], have revealed that there are time-of-day differences in melanopsin-mediated pupil responses [14, 15]. While these studies were not designed to differentiate between circadian and homeostatic effects and quantify isolated effects of the different post-receptoral retinal mechanisms, they suggest, indicating that at least some variability originates in the retina."*

[R1.3] In general, the captions' figures need to contain a more detailed description.

We thank the reviewer for their comments. We have revised the figure captions to be longer and more informative.

[R1.4] Although the tradition in vision science is that rods are saturated in photopic light levels, as the authors pointed out (Line 405), there is new evidence that rods might signal at high light levels (see Uprety et al., iScience, 2022). I think it is worth conducting a control experiment to discard rod intrusion in the planned experimental conditions or to better explain why rods would not affect the results of the planned study.

We recognize the possibility of rod intrusion in our nominally photopic stimuli. Based on the evidence of rod escaping saturation at different light intensities and temporal

frequencies, our experiments may be differently affected. We agree with the reviewer, and we will report the results of post-hoc experiments with the publications of the psychophysical and pupillometric results, respectively, as described in the section “Control for rod intrusion”. In addition, we will also carefully and transparently describe calculations that relate the background light level of our stimuli to estimates of rod intrusion in the literature.

[R1.5] The effect of tiredness in psychophysical tasks and Pupillary recordings is well known. The authors have included an alertness and vigilance procedure to control this effect. However, I wonder what would be the exclusion procedure in this case. If the participants fail this test, then the complete measurement is excluded? I guess that the last sessions might be very demanding. The authors might want to explain a little bit more this procedure.

The participant management in case of measurement failure due to tiredness or non-compliance has now been described in the section "Study procedure”*:*

> “*The experimenter will continuously monitor compliance and the successful completion of experiments. Given the demanding nature of the protocol, there is tolerance for participant's failure to perform individual tests. A block's data is considered for analysis as long as one test repetition within that block is successful.*
>
> *However, experimentation will proceed across all modalities using light-emitting devices, even if data collection issues occur in a single modality. These devices serve a dual purpose: not only do they measure visual outcomes, but they also provide consistent light exposure > 10 lux which is important for maintaining comparability between participants.*
>
> *If the experimenter fails to collect data from both primary modalities - pupillometry and psychophysics - for more than one block due to any reason (e.g., non-compliance, fatigue, tiredness), the experimental session may be aborted. This is because the increased measurement intervals would render the data unsuitable for testing circadian hypotheses.”*

[R1.6] Section 1.4: Modulation of the cone-opponent process L-M in pupillometry. I wonder If the authors think that this hypothesis can provide information about the M-cone out-of-phase response found by (Woelders et al, PNAS, 2019, and Murray et al., JOSA A, 2019), which is not in agreement with a previous study (Barrionuevo and Cao, JoV, 2016) and the physiological evidence provided by Dacey and colleagues (Nature, 2005)?

We are aware that the two existing papers found an out-of-phase M cone response, adding to the prior findings of S cone opponency in the pupil response (in line with Dacey's findings from primary ipRGCs). Our study did not set out to examine this particular M cone response, but should our results inform this question, we will report any findings.

[R1.7] Line 57-58: Maybe I'm not understanding well but it seems like a circular rationale to me.

We've extended the paragraph by another sentence making the idea of a closed loop system clearer. The limit oscillator models provide a theoretical framework for the pacemaker's phase response curve and fit the empirical data. Nevertheless, it is worth investigating the biological substrate of the "front end" of the circadian system, i.e., the spinothalamic tract, to better understand the dynamics of the input and output of the pacemaker. We believe that circadian variations in sensitivity are likely given existing results about systematic (diurnal) variation in many other physiological structures, tissues, and organs.

[R1.8] Fig. 1: Is Actigraphy conducted for 20 days, 21 days, or more (considering the figure)?

Actigraphy measures were taken out from Figure 1. We realized that mixing labels for stages of the experiment, and (part of the) experimental outcomes can lead to confusion. Actigraphy will be employed to confirm adherence to sleep and wake times throughout all circadian stabilisation period, lasting 21 days (3x 7 days).

[R1.9] Line 300-302: glaucoma may cause disruptions to sleep (Gracitelli et al., IOVS, 2014), so the authors might want to explain more about it and the reason for this exclusion criteria.

We thank the reviewer for this comment. We have elaborated on the criteria as follows:

> "*An ophthalmological exam including fundus photography and Optical Tomography images (macular image and optic disc image) will take place to confirm ocular and retinal health. Our primary concern is to exclude individuals with any ocular disease or compromised visual information transmission, such as in glaucoma patients. A cup-to-disc ratio of > 0.3 will lead to the exclusion of the participant, as well as an intra-ocular pressure (IOP) > 20 mmHg. Criteria are based on the European Guidelines for glaucoma diagnostics by the European Glaucoma Society to exclude participants with potentially diseased eyes [36].*

These criteria may be conservative as a higher cup-to-disc ratio does not necessarily indicate disease, especially for individuals with larger papilla size, as then, automatically, the physiological papilla excavation increases. However, these criteria can be applied algorithmically, facilitating a quicker decision without additional consultation."

[R1.10] Line 505: type "as" should be "has"

Thank you for pointing out this typo, which we have fixed.

Reviewer 2 comments

[R2.1] 1. Rationale: The umbrella study question is fantastic and very timely. The one area that needs more justification is using the silent substitution method. Until that is the dominant method and is validated against other more commonly employed methods (which arguably could be done here), it needs additional justification.

The stimulation method compared to the stimulation with single- or narrow-wavelength stimuli is now discussed as part of the comparison of PLR and PIPR outcomes.

[R2.2] Add rationale for light exposure midway through the protocol. Is this expected to shift the clock or not, and what about its timing is intentional and why? Specifically, a clearer description of how the data from this visit will be compared with the FD protocol data, i.e., how will this visit inform whether the tasks in the FD elicited a phase shift? Also, it is likely some measurements, particularly phase markers, will have been influenced by the first week of the stabilization period. Please include a rationale for including this study visit at this particular time point (e.g., halfway through the stabilization) and not at a more representative baseline. Please also clarify whether this light exposure session mimics one cycle of tasks completed during the FD and if these will be conducted under the same lighting conditions as the FD.

We clarify the points as follows:

- Participants start stabilisation one week prior to the experiment. Additionally, we remind participants 3 days before their experiment starts to adhere to their self-selected sleep schedule.

- Influence delay in sleep time on the circadian system: We assume that a delay in bedtime by one hour due to the necessity of melatonin measurements past bedtime may disturb the circadian system. However, the distance to the next experimental session should be sufficient for participants to go back to their usual rhythm.
- Influence of light exposure & rationale of light exposure session: We assume that the evening light exposure (representative of the experimental light exposure during the 40h protocol) is not strong enough to affect the circadian pacemaker. In this protocol, the main goal is to estimate circadian rhythmicity – possibly controlled by the SCN – in terms of visual functions. The circularity concern referred to in the paragraph "Q*uantification of an effect of the protocol on the circadian system"*: if the light exposure that necessarily has to be applied to measure visual outcomes exerts an advancing or delaying effect on the circadian measurement (visual system → circadian pacemaker), but we assume a directed effect only from the circadian pacemaker on visual functions (main hypotheses: circadian pacemaker → visual system), this would pose a problem of circularity. We wouldn't be able to isolate the pure effect of the circadian pacemaker on the visual system. As this is a dilemma that does not have a solution due to the nature of visual experiments, we quantify the effect of the experimental light exposure on the circadian system to report and estimate the extent of the effect, if present at all.

[R2.3] Protocol: Please justify how sleep homeostasis will not vary across the 40-hour protocol so as to remove its effect and allow the circadian effect to be disentangled. If it begins upon wake, process S will be low and sleep may not occur in the first few sleep opportunities, although process S will accumulate as the morning process C increase in alertness rises. If sleep cannot occur until the 3rd or 4th sleep opportunity, please explain how process S will be kept stable so as to isolate process C.

We thank the reviewer for this comment. We have added another reference of the study/studies who first established the protocol in 2004 (Münch et al. 2005, Eur. J. Neurosci.; Graw et al. 2004; Physiology & Behavior), referring to cycle length, sleep-to-wake ratio and a preceding adaptation night to start out with low sleep pressure. Both studies were done on both sexes, part of their sample being from the same demographic (20-31 years). The reviewer voices valid concerns about the feasibility of the study. We have contacted the authors about the study's feasibility and success rate of sleep during circadian morning and noon. Based on their reports and pilot testing of the sleep schedule in our laboratory, we are confident to successfully establish a nap protocol.

However, nap periods are essential for preventing the accumulation of sleep pressure throughout the circadian day. We are continuously tracking people's self-reported sleep efficiency. If they are unable to sleep for two naps, we will discontinue the experiment of this individual, as by that time, sleep pressure has accumulated over 7.5 hours.

Our protocol is a compromise between the 20/28-hour "forced desynchrony" protocols used to most reliably decouple circadian factors and the sleep-wake cycle and the "constant routine protocol," which would bear the problem of confounding effects of sleep pressure visual or neurobehavioral outcomes. The assumption of sleep pressure to be low is supported by evidence from psychomotor vigilance testing, where typical fatigue-related deterioration of performance (slowest RT) was found in the sleep deprivation condition but not in the nap condition that uses the same protocol as the present study (Münch et al. 2005, Eur. J. Neurosci.)

[R2.4] Please explain how the PLR will address non-image forming photoreception, as PIPR methods are more typically used for this with post-illumination measurement. Please provide additional justifications for pupillometry methodological decisions.

First, it is unclear why only acute pupillary responses are being investigated, given the proposed ability to isolate and capture melanopsin-driven responses to light. Given recent interest in the role of the melanopsin system in circadian photoentrainment, it seems remiss to not measure the post illumination responses of the melanopsin system to light which has been used more than the silent substitution methods. At the very least, both could be included since pupillometry has already been incorporated.

The pupillary response to light to silent-substitution stimuli can be used to measure melanopsin sensitivity, providing additional information compared to the PIPR method, which relies on monochromatic stimuli, leading to a differential redilation rate. Spitschan et al. 2014 (PNAS) described the temporal bandpass properties of the pupil control to chromatic, melanopic, and luminance stimuli, which we used to inform the selected temporal frequency of our stimuli. At the temporal frequency, we expect good response amplitudes to all proposed conditions. Due to limitations in our protocol, we were unable to add additional pupil measures.

[R2.5] Further, while standardizing entrance pupil diameter constrains retinal irradiance, it also removes potentially meaningful individual differences in responses to light. Please include a justification for this methodological decision

and the consequences of not using pharmacological dilation and consensual response or the Maxwellian view, for example.

The Maxwellian view approach uses an entrance pupil that is smaller than the naturally occurring pupil. This has the benefit that any variations in participant pupil size do not affect the measurements. The logic here is that we are not interested in variations in psychophysical performance that are due to mere variations in retinal irradiance arising indirectly from differences in pupil size (which do occur) but reflect true variability in neural processing.

[R2.6] Finally, please report how the dominant eye will be determined and why this is necessary.

We've added a sentence to the in-person screening description: "*Selecting the dominant eye for monocular viewing conditions."*

We are using monocular viewing conditions for the purpose of control – there are various processes that could influence eye dominance and could thereby change the contributions of each eye to the visual performance (e.g., fatigue or eye strain). We select the dominant eye to, first, standardize the selection of the eye across participants and, second, to reduce vision-related fatigue.

[R2.7] Language - there are a few typos and confusing sentences that can be easily edited. These include "modulation of alertness of light" by which the authors might mean "modulation by light of alertness." The sentence "Studies investigating how the 58 pupillary light reflex, which reflects the response of all photoreceptors to light [12,13], have revealed that there are time-of-day differences in melanopsin-mediated pupil responses [14,15], indicating that at least some variability originates in the retina" could be revised. There is a typo somewhere in "...and to create regular cycles of sleep and wakefulness." An exhaustive list of possible edits hasn't been detailed here, so please review and edit carefully to ensure clarity.

Thank you for pointing out these typographic mistakes. We have corrected them.

[R2.8] In addition, the PLR and PIPR are distinct, and driven by different photoreceptor classes. It is not clear here how the time at which pupil diameter is measured correlates with the photoreceptors that are signaling, and how the PIPR differs in it's ability to reflect ipRGCs. Naturally, the rods and cones project to ipRGCs, but only ipRGCs are signaling in the post-illumination period. Until

silent substitution measures are validated and standardized, including the PIPR allows researchers to balance the pros and cons of both.

Research on PIPR has been important in informing our hypothesis. However, we selected the PLR as opposed to the PIPR metrics for various reasons: first and foremost, the time efficiency of the experimental design. The restriction of experiment time during wake time in each block conflicts with the necessity of dark adaptation during PIPR protocols.

Moreover, there is research about photoreceptor contribution to different dynamics in pupillary control, e.g. cones are especially relevant in the initial constriction, while melanopsin is more relevant in the sustained pupil constriction of later redilatation. However, melanopsin does contribute to the PLR being activated in the optimal temporal frequency (more details above). We are confident that we will achieve a response amplitude that is large enough. Generally, we see the problem that rod signaling would have a considerable influence on the PIPR and will, in turn, address questions of rod saturation in our protocol.

[R2.9] Please specify inclusionary/exclusionary SRI cutoffs.

The Sleep Regularity Index (SRI) will not be used to include or exclude participants. It will only be considered to establish a priority order among participants if a selection is needed due to limited time and room capacity.

[R2.10] In the metabolic processes section: 14 days should be after light exposure session through 1 week after FD based on study protocol diagram not until after the FD session.

Thank you for pointing out this mistake. We have further taken the chance to adapt the figures in order to increase clarity. Figure 1 now only includes timing of experimental session and stabilisation periods without further specifying measurement modalities; Figure 2 now includes all measurements. Data collection duration for continuous measurements during circadian stabilisation is only described in the text [MOU1]to avoid confusion:

> "*We will monitor metabolic activity starting the morning after the light exposure session for a total of 14 days, thereby extending into the stabilisation period after the forced-desynchrony session (post-FD stabilisation)*."

[R2.11] It may also be worthwhile to further explain how light history information from the circadian stabilization phase will be used to inform the FD pupillometry data.

We are only aware that there are correlations between the PIPR and long-term (> few hours) light exposure measured by Actiwatch in children (Ostrin, 2018; Ophthalmic and Physiological Optics), possibly a proxy for habitual light exposure. Yet, we are not aware of any predictable influence of the long-term history of light exposure on the PLR or the pupil response in general. Actigraphy data is primarily used to understand compliance with the self-selected sleep schedule during stabilisation, and will not be added to the statistical models during confirmatory hypothesis tests. By the experimental design, we make sure that participants have spent about 15 hours before the experimental light exposure of the forced-desynchrony study starts, under controlled, dim light (thus comparable) conditions. We may elect to perform exploratory analyses incorporating light exposure.

[R2.12] Please more clearly lay out the importance and difference of the contrast sensitivity and silent substitution methods and what they measure that is relevant to sleep and circadian rhythms. Visual function is not thought to vary, but perhaps this is of interest somehow?

Contrast sensitivity is only relevant for the psychophysical measurement. We add a sentence to the paragraph "Postreceptoral temporal contrast sensitivity," explaining: "*For statistical modeling, we will use the contrast sensitivity, which is the inverse of the contrast threshold.*"

The goal of silent substitution is – by carefully tuning the light spectra to modulate the activity of one or more specific photoreceptors while keeping the response of other selected photoreceptors constant (Estévez & Spekreijse, 1982; Vision Res). For example, in the "Mel" condition in the pupillometry design, during visual stimulation, cones respond to the same extent while only melanopsin is activated. Therefore, ipRGC signaling is thought to only be caused by melanopsin activation but not by cone input.

Regarding our interest in variation in visual function, we have extended the introduction to make our motivation clearer. In summary, there is already evidence of diurnal variation in visual functions, but more specific methods should be used in order to confirm results for the postreceptoral mechanisms, which correspond closer to the physiological structures. Yet, restricting measurements to below 24 hours and without a control for sleep pressure does not allow conclusions of circadian effects. Thus, the origin of modulation is still unclear – a gap that the present study will fill.

[R2.13] The terms used in the field typically segregate into PLR for pupil light reflex, first reported by Lowenfeld in 1999, and PIPR for post-illumination pupil response, described in 2007 by Gamlin. If this is not the right terminology to be used, please clarify for readers so that the different goals of the PLR and PIPR are not confused and are more clear. Given that the PLR is often in response to light but also images or sound, I think it is too broad to be used to describe ipRGC driven responses to chromatic stimuli and could be confused. How will you distinguish this? Is there a reason the post-illumination period is not preferred?

We address the points as follows:

- Distinguish between PLR and PIPR: We have added a paragraph in the discussion ("Selection of pupillary response metrics") to inform the reader about the difference between PLR and PIPR.
- Multisensory sensitivity of the PLR: It is indeed known that the PRL can be modulated by auditory or emotive stimulation. This has been considered in the experimental design: for the stimulation, participants enter a 1.3m x 1.3m x 2.2m tent. The participant will not be able to listen to any audio and is instructed not to speak to the experimenter. While it is not soundproof or echo-free, as a context cue, the entrance into another "room" promotes a behavioral modification.
- Reasons for not including the PIPR: In the current protocol, it is infeasible to add the measures of the PIPR given the current study design. The PIPR measurements require an extension by a dark adaptation time, extending the protocol by at least multiple minutes. The test would exceed the available time given that the Experimenter also needs to align the Maxwellian view system to the participant every time. As shown in Figure 3, containing the experimental schedule per block, the experiments are tightly scheduled, and the pupillometry protocol does not allow include longer trials,

[R2.13] Adding the PIPR to the protocol would be a great service to the field.

We agree but are unable to include it in this specific study.

[R2.14] Please explain why exactly the Zele and Münch papers on diurnal variation in PIPR from about 2009 are not sufficient for your purposes, and specifically, what they might have misrepresented by not employing FD measures. Is process S thought to affect the PIPR? Is sleep homeostasis known to impact ipRGCs? Theoretically, is there a rationale for why the Zele and Münch papers are unlikely to reflect the daily variation in ipRGC responsivity?

It is currently unclear whether the variations in color and luminance perception can specifically attributed to varying cone sensitivity. Furthermore, without empirical testing that at least keeps homeostatic influences constant, diurnal variation cannot with certainty be attributed to circadian processes, which would be an important step for a mechanistic explanation of this variation. It is currently not known whether ipRGCs are affected by process S, but based on the current literature, it can neither be confirmed nor rejected and is worth investigating for the purpose of fundamental research but also for clinical applications.
